# Wavelength-tunable passively mode-locked mid-infrared Er^3+^-doped ZBLAN fiber laser

**DOI:** 10.1038/s41598-017-13089-6

**Published:** 2017-11-02

**Authors:** Yanlong Shen, Yishan Wang, Hongwei Chen, Kunpeng Luan, Mengmeng Tao, Jinhai Si

**Affiliations:** 1 State Key Laboratory of Transient Optics and Photonics, Xi’an Institute of Optics and Precision Mechanics, Chinese Academy of Sciences, Xi’an, Shaanxi 710119 China; 2 Shaanxi Key Laboratory of Photonics Technology for Information, School of Electronics and Information Engineering, Xi’an Jiaotong University, Xi’an, Shaanxi 710049 China; 3 State Key Laboratory of Laser Interaction with Matter, Northwest Institute of Nuclear Technology, Xi’an, Shaanxi 710024 China; 4 University of Chinese Academy of Sciences, Beijing, 100049 China; 5 Collaborative Innovation Center of Extreme Optics, Shanxi University, Taiyuan, Shanxi 030006 China

## Abstract

A passively mode-locked Er^3+^-doped ZBLAN fiber laser around 3 μm with a wide wavelength tuning range is proposed and demonstrated. The laser cavity was comprised of a semiconductor saturable absorber mirror and a blazed grating to provide a wavelength tunable feedback. The central wavelength of the mode-locked fiber laser can be continuously tuned from 2710 to 2820 nm. The pulse train had a maximum average power of higher than 203 mW, a repetition rate of 28.9 MHz and a pulse duration of 6.4 ps, yielding a peak power of exceeding 1.1 kW. To the best of our knowledge, this is the first demonstration of a wavelength-tunable passively mode-locked mid-infrared fiber laser at 3 μm.

## Introduction

Passively mode-locked mid-infrared (mid-IR) lasers emitting near 3 μm waveband, featured with high peak power and short pulse duration, have gained increasing research interests owing to their promising applications including laser surgery, spectroscopy, material processing and mid-IR supercontinuum source pumping^1–4^. Compared with other lasers, fiber lasers have a number of inherent advantages, such as high stability, excellent beam quality, compactness and tunable output^5^. Therefore, passively mode-locked mid-IR fiber lasers have been widely investigated based on Er^3+^ or Ho^3+^ doped fluoride fibers during the past few years.

Currently, the reported ~3 µm passively mode-locked fiber lasers can be roughly divided into two major categories. The first one is nonlinear polarization effect, i.e., nonlinear polarization rotation (NPR) in ring cavities. Duval *et al*. reported an ultrashort Er^3+^-doped fluoride glass fiber ring laser using NPR with an average output power of 44 mW and a pulse duration of 207 fs, which was the first demonstration of passively mode-locked fiber laser in femtosecond scale at this waveband^1^. Antipov *et al*. demonstrated the generation of a high peak power of up to 37 kW laser pulses with a pulse duration of 180 fs from a 2.9 μm passively mode-locked Ho^3+^/Pr^3+^-codoped fluoride fiber ring laser by employing the same technique very recently^2^. The second one is introducing saturable absorbers (SAs) such as Fe^2+^:ZnSe crystal, InAs, and semiconductor saturable absorber mirror (SESAM) into cavities. Wei *et al*. reported a passively mode-locked fiber laser based on a Fe^2+^:ZnSe crystal^6^. Hu *et al*. obtained passively mode-locked pulses with an average output power of 69.2 mW and a pulse duration of 6 ps from a Ho^3+^/Pr^3+^ co-doped ZBLAN fiber laser by inserting a SA of InAs in the ring cavity^7^. Compared with these SAs, SESAM, as a type of mature commercial SA, has been extensively adopted to attain stable passive mode-locking due to its excellent properties as well as the ability of customizing some of its parameters e.g., modulation depth, saturation fluence, recovery time, etc^8^. Li *et al*. have reported the passive mode-locking of a Ho^3+^/Pr^3+^-codoped fluoride fiber laser using a SESAM^9^. Tang *et al*. scaled the average output power significantly up to 1.05 W from a stable high-average-power passively continuous-wave mode-locked (CML) Er^3+^-doped ZBLAN fiber laser at 2.8 μm with a pulse duration of 25 ps very recently^4^. Benefitting from the high gain of high concentration Er^3+^ or Ho^3+^ ions doped fluoride fibers, the fiber end facet with feedback of ~4% was allowed to be commonly used as the output coupler in previous configurations of^6^ and^9^. However, Habucha *et al*. thought it difficult to obtain a stable CML operation in these cavities due to the weak and broadband cavity optical feedback provided solely by Fresnel reflection from the fiber end facet^10^. Based on the approach of inserting a grating into cavity that provides higher, controlled and spectrally selective reflection, the CML was easily obtained and the long-term stability was excellent. They hence chose a higher reflection fiber Bragg grating (FBG) as the output coupler to achieve a long-term stable CML operation with an average output power of 440 mW^3^. Nevertheless, FBG is hard to fabricate in fluoride fibers, and consequently is not easily available. In particular, the tunability of FBG is limited^11^. Since mode-locked fiber lasers with wavelength-tunable output emitting in the mid-infrared region could offer possibilities in applications such as frequency comb generation^12^, spectroscopic sensing^13^, mid-infrared microcavity lasers^14^ and especially pumping sources for other nonlinear optical systems^15^, it is still highly encouraged to explore a wavelength tunable CML fiber laser at mid-infrared wavelengths. A possible solution is introducing a blazed grating as the cavity feedback, which has been widely used in Er^3+^ and Ho^3+^ doped fluoride fiber lasers operating in the continuous-wave regime^16,17^ and the Q-switching^8,15^ with a huge tuning range. However, until now, there are no investigations on wavelength tuning properties of the 3 μm Mid-IR mode-locked fiber lasers.

In this paper, we proposed and demonstrated for the first time a wavelength-tunable passively mode-locked Er^3+^-doped fluoride fiber laser to the best of our knowledge. Combining a SESAM as the absorber and a blazed grating as the wavelength selective feedback, the laser produced CML pulses with a pulse duration of around 6 ps and a wavelength tuning range of over 100 nm from 2710 to 2820 nm.

## Results

**Figure 1:**
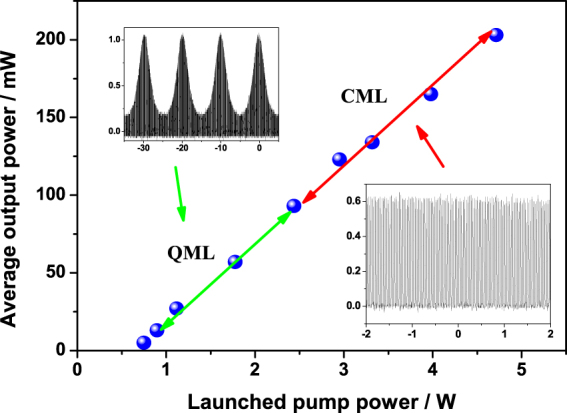
Average output power as a function of launched pump power. Inset: The two operation regimes at different output powers.

At the beginning, we adjusted the grating to maximize the output power of the fiber laser under a certain pump of slightly higher than the threshold of ~0.7 W. The laser output power was measured with a thermal powermeter (Gentec, XLP12-3S-H2-D0). The mode-locked pulse train was simultaneously detected with an HgCdTe detector (Vigo PVM-2TE-10.6, rise time < 3 ns) and monitored with a 1 GHz digital oscilloscope. As increasing the pump power, the fiber laser went through three stages, i.e., self-pulsing (pump < 0.8 W), stable Q-switched mode-locking (QML) and continuous-wave mode-locking (CML), which was analogous to the results in^3^. Average output power at various stages versus launched pump power is plotted in Fig. 1. The laser operated in the QML regime for incident pump powers ranging between 0.8 and 2.4 W, and switched to the self-started pure CML regime when the pump power was beyond 2.4 W. This CML operation was sustained up to the maximum pump power of 4.7 W, corresponding to the maximum average output power of 203 mW, and the corresponding average power from output 2 was around 81 mW. We did not try with higher pump power because increasing the pump power ran the risk of damaging the SESAM, even though the CML state was still maintained when further increasing the incident pump power.

**Figure 2:**
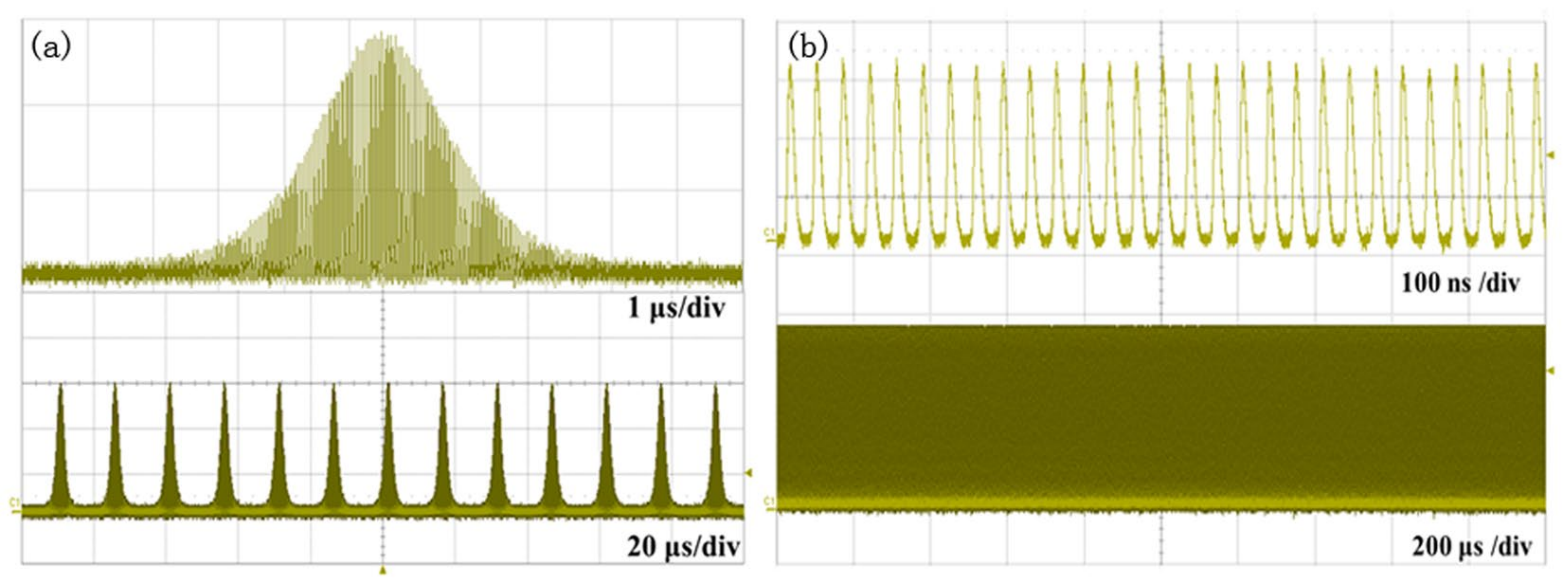
Measured pulse trains in different time scales recorded at two states of (**a**) the QML state under pump power of 1.7 W and (**b**) the CML state under the maximum pump power of 4.7 W.

Figure 2 shows the QML and CML pulse trains measured in different time scales. As expected, the pulse envelope amplitude decreased gradually and the repetition rate of the pulse envelope increased as increasing the pump power. It can be seen from Fig. 2(a) that the stability of the QML pulses with little fluctuation was fairly high. The marvelous regularity without any envelope modulation of the pulse trains in Fig. 2(b) confirms the CML operation. The period of the pulse train was about 34.6 ns, which was consistent with the round-trip time of the laser cavity. Long-term stability of the mode-locking operation at the maximum output power was monitored over 1 hour and the regular pulse trains were always kept.

**Figure 3:**
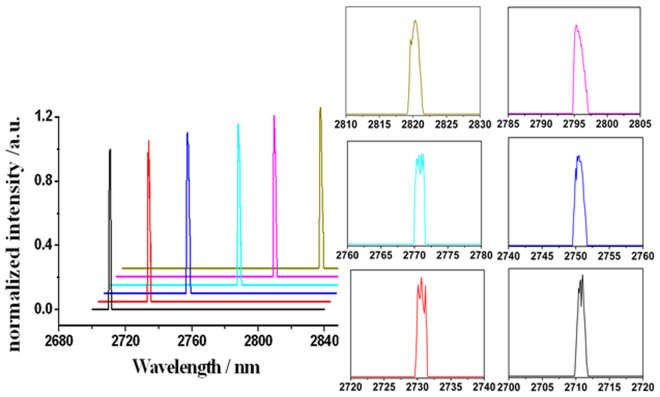
The typical tunable spectra of the laser output in the CML regime.

The laser tuning spectra in the CML regimes were recorded by an optical spectrum analyzer (Andor Shamrock 750) with a resolution of 0.1 nm, as shown in Fig. 3. The FHWM linewidths of the laser pulses were kept around 1.5 nm during the entire tuning range.

**Figure 4:**
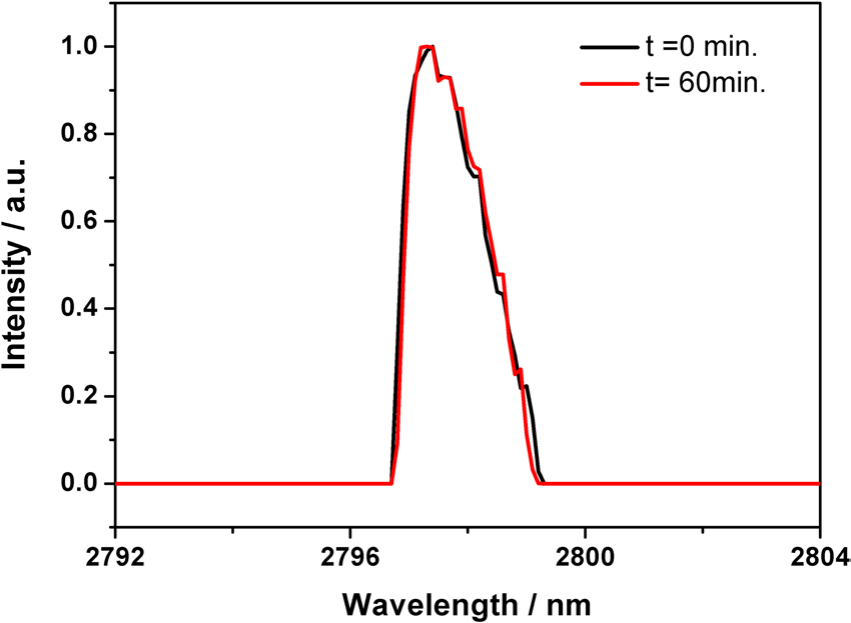
Measured spectra of the CML at 2797 nm before and after one hour operation.

Fixing the grating at a certain position, the output spectra were measured for a period of one hour, as typically shown in Fig. 4. The central wavelength of the CML output remained essentially unchanged after one hour operation.

**Figure 5:**
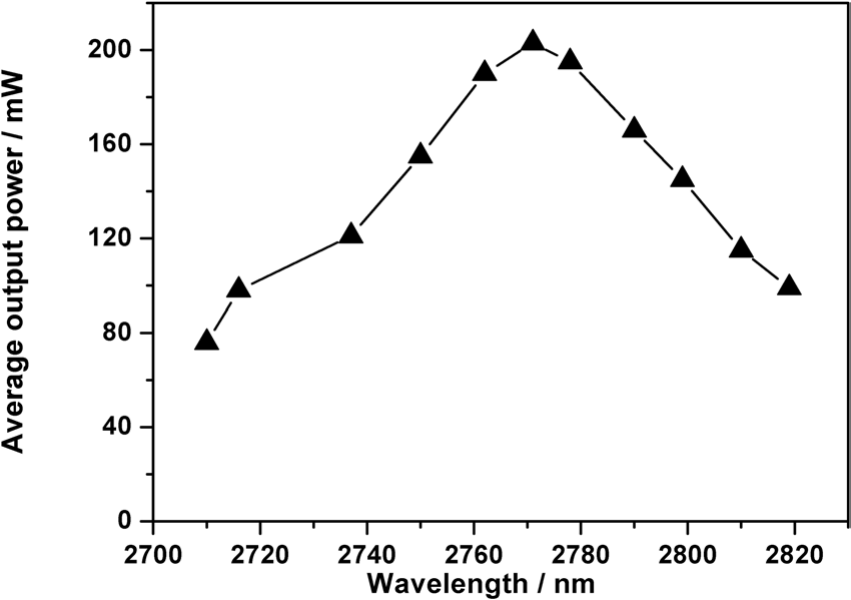
Average output power in the CML regime at various operating wavelengths under the maximum pump power of 4.7 W.

In the situation of the fixed maximum launched pump power of 4.7 W, the output power in the CML regime as a function of tuning wavelength is shown in Fig. 5. Similar to the tuning curve in the CW regime, the tuning range was at least over 100 nm, the average output power increased initially and then decreased at the long wavelength range, which matches well with the intra-cavity gain profile in^18^.

**Figure 6:**
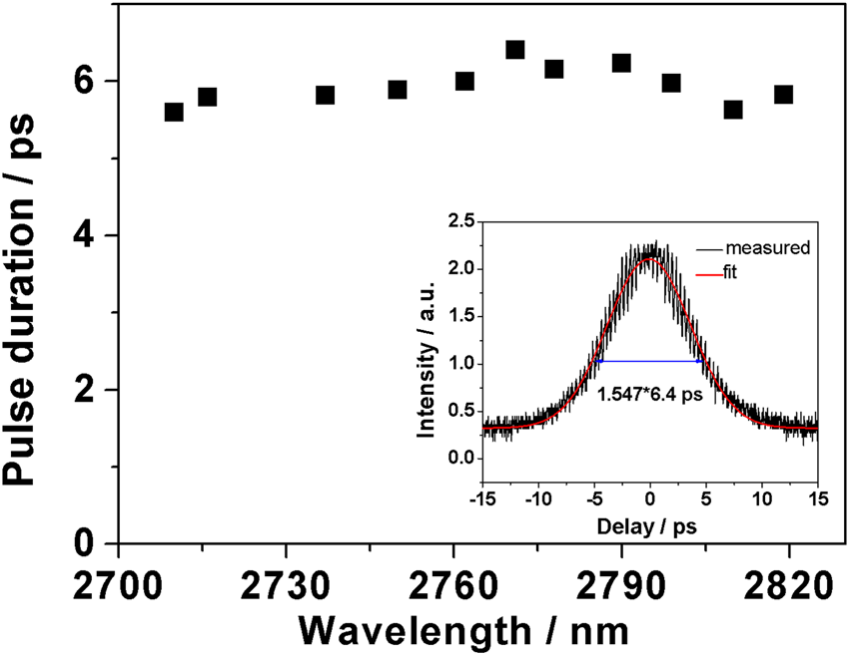
Pulse duration in the CML regime as a function of wavelength. Inset: Autocorrelation trace of the mode-locked pulses measured at the maximum output power.

The mode-locked pulse duration at different wavelengths was measured using a commercial autocorrelator (APE pulseCheck USB MIR), as typically shown in Fig. 6. The pulse duration varied in the range of 5.6~6.4 ps when tuning the laser. The trend matched well with the calculation result in^19^ that the larger the small signal gain, the longer the pulse duration. The inset is the autocorrelation trace of the mode-locked pulses measured at the maximum output power. Assuming the CML pulses are sech^2^-shaped, the measurements suggested a pulse duration of 6.4 ps. Consequently, the peak power was calculated to be about 1.1 kW at the maximum output power. The associated time–bandwidth product of the CML pulses was 0.38, indicating that the pulses were near to but not transform limited and were chirped. The ZBLAN fiber natural dispersion and mode dispersion mainly led to the chirped pulse.

**Figure 7:**
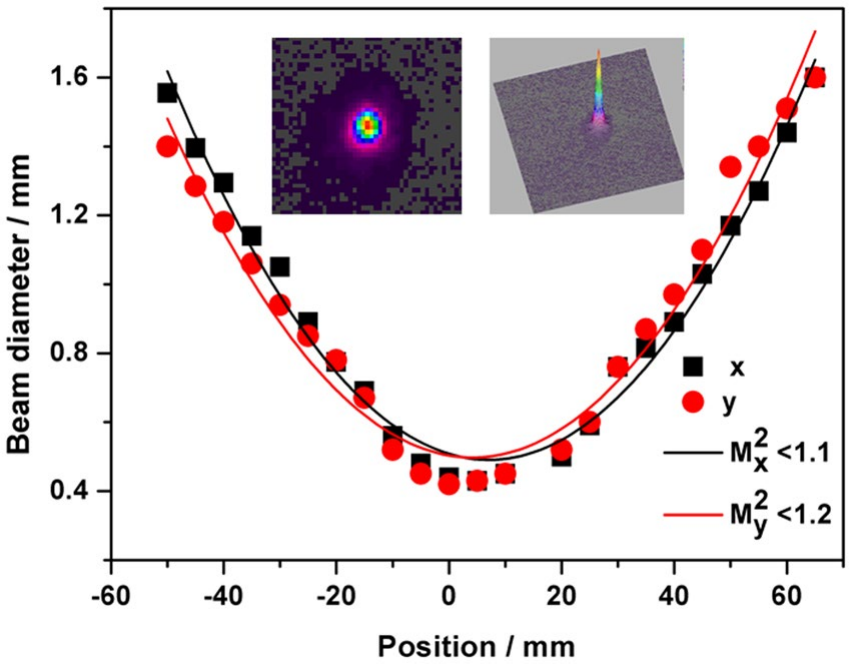
Laser beam diameters as a function of distance from the waist location (where z = 0). Inset: Far-field images of the laser beam displayed in 2-D and 3-D, respectively.

The ISO-standard caustic measurements was performed to measure the laser output beam quality factor *M* ^2^ with a mid-infrared camera (OPHIR, PYROCAM III), and the results are shown in Fig. 7. The far-field beam spot had a symmetrical Gaussian distribution, and the beam quality *M* ^2^ was calculated to be less than 1.1 and 1.2 for x-axis and y-axis, respectively, giving the *M* ^2^_eff_ of around 1.15^20^.

## Discussion

The slop efficiency in our experiment was determined to be around 7%, which was much lower than the result in^4^. Two principal factors might be responsible for such low efficiency. On one hand, the output coupling ratio (~77%) was much lower than that (~96%) in^4^. On the other hand, there was some reflective loss of the grating and the SESAM. Furthermore, the introduced insertion loss of the three uncoated CaF_2_ lenses in the cavity and the coupling loss of both fiber ends also played a key role in limiting the efficiency. And thus, choosing the inserted lenses with anti-reflection coatings will be beneficial to improving the efficiency.

With respected to the spectra in Fig. 3, it can be seen that there are some ripples in the top of the spectra when the operating wavelengths were shorter than 2770 nm, and the ripple almost disappeared as tuning the wavelengths to longer than 2790 nm. It is thought that the atmospheric water vapor absorption lines lead to the ripples because the vapor absorption lines at the wavelength range of 2710 ~ 2770 nm are much stronger than the wavelength beyond 2770 nm according to the HITRAN database^21^. The central wavelength at different stages including the QML and CML regimes by increasing the pump power was quite stable when fixing the grating due to the wavelength-locking effect of the grating^22^.

Compared with the tuning curves in the CW regime, the tuning range in^16^ was slightly narrower than in^17^. Under the same pump level of around 5 W, the tuning range in^17^ was 2700–2840 nm, while the tuning range of CML operation in our case was 2710–2820 nm (under the pump power of 4.7 W, the parameters of our fiber close to^17^). Beyond this range, the laser signal could still be detected, nevertheless, the pulse trains of the mode-locking fluctuated dramatically.

There is a background signal of the autocorrelation trace. A possible origin of this was the generation of the partial Noise-like-pulse (NLP) operation, which is characterized by the circulation of an optical noise burst and widely studied in Er^3+^ ^23^ and Tm^3+^-doped silica fiber lasers^24^.

The limited soliton pulse energy before pulse breaking or multi-pulsing occurring can be calculated by the equation below^25^:

1 $${E}_{soliton}=\frac{3.11{\lambda }^{2}| {D}_{ave}| }{2\pi c\gamma {\tau }_{FWHM}}$$

where *c* is the speed of light in vacuum, the central wavelength λ and the pulse duration *τ*_FWHM_ were measured to be 2780 nm and 6.4 ps in our case. The nonlinear coefficient γ can also be calculated to be 3.9 × 10^−5^ W/m(assuming the nonlinear refractive index is 2.1 × 10^−20^ m^2^/W for fluoride fiber^26^), and the average dispersion of the cavity *D*_ave_ could be determined to be −6.99 ps/nm/km using the typical formula 2πc|β_2_|/λ^2^. The calculated limited soliton pulse energy was ~1.07 nJ, which was lower than the experiment result of ~7.02 nJ.

The optical spectrum of NLP operation is typically wideband, from 15 to 150 nm,^27^, which involves the accumulation of a large nonlinear phase shift per cavity round trip. In contrast to standard mode locking, the circulating pulse has a fluctuating internal structure. However, the linewidth in our case was much narrower than the pure NLP operation. It was thought that the operation of the laser balanced between the standard mode-locking and NLP operation. And thus the partial NLP introduced background into the autocorrelation trace, which reflected the temporal extension of the noise burst^28^.

It should be pointed out that the active fibers of all the previous reported mode-locked mid-infrared ZBLAN fiber lasers were single-mode at 2.8 μm^1–4,6,7,9^. Whereas the active fiber used in our mode-locked cavity has a larger core size and the V number of around 4.44, which means that the fiber is multimode. Generally, large core could lower the possibility of nonlinear effect in the fiber, and guarantee the power scaling ability of the fiber laser as well^4^. However, it’s difficult to obtain CML in multi-mode fiber due to the fact that mode dispersion normally prevents the laser from generating short pulses. This interesting behavior of the CML in the multi-mode fiber could be probably explained by the fact that most of the laser signal was constrained within the fundamental mode by the feedback of the SESAM or the blazed grating.

Considering the time-bandwidth limit that the broader the spectrum, the narrower the pulse duration, indeed, there was no advantage in the pulse duration because of the narrow linewidth when using grating as the feedback. However, based on the fact that the grating can provide controlled and spectrally selective reflection, we believe the high feedback and wavelength selective effect of the grating play a key role in achieving the CML operation. On one hand, unlike redshift of spectra in previous demonstrations, the spectra of this CML laser were quite stable due to the locking effect of the grating, which in turn improved the stability of the CML pulses. On the other hand, to obtain CML operation, the in-cavity pulse energy should be beyond Eth, which is expressed in Eq. (2)^10^.

2 $${E}_{th}^{2}={E}_{sat,g}{E}_{sat,a}{\rm{\Delta }}R$$

where *E*_*sat,g*_ is the saturation energy of the gain medium, *E*_*sat,a*_ is the saturation energy of the saturable absorber (product of saturation fluence and effective beam spot on the absorber, and Δ*R* is the modulation depth (18% in our case). In order to lower the energy threshold, a SESAM with a lower saturation fluence and smaller modulation depth should be chosen. However, for a certain laser cavity consisted of the fixed gain fiber and SA, the threshold is determined. Therefore, as mentioned before, improving the feedback, i.e., decreasing the output portion of the output coupler is an effective approach to increase the in-cavity pulse energy to meet the threshold.

The laser can be used to deliver an ideal wavelength-tunable ultrashort pulsed seed for amplification in this waveband to act as a pump source for supercontinuum generation covering the whole mid-IR. Furthermore, it is convenient to replace the SESAM with other SAs by using the current laser configuration, and thus we could also compare the properties of these different SAs.

## Methods

**Figure 8:**
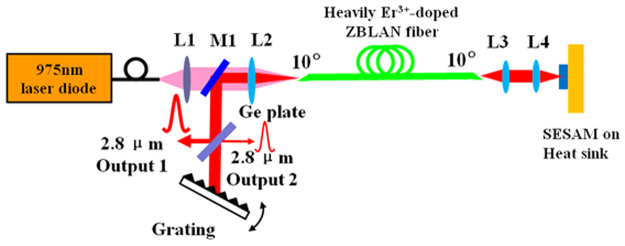
Experimental setup of the wavelength-tunable passively mode-locked Er^3+^-doped ZBLAN fiber laser. (M1: dichroic mirror, L1: collimator, L2, L3, L4: CaF_2_ lenses).

The experimental setup of the tunable SESAM-based mode-locked fiber laser is schematically depicted in Fig. 8. The pump source is a 27-W fiber-coupled laser diode centered at 975 nm. The active fiber was a piece of 3.36 m heavily (concentration of 6 mol%) Er^3+^-doped ZBLAN multimode core double-clad fiber provided by FiberLabs. The core diameter and NA are 33 μm and 0.12 respectively, while the inner cladding has a diameter of 330 μm and a NA of 0.55. Unlike configurations in most previous reports that the laser cavity output port was the active fiber end facet, both ends of our fiber were cleaved at an angle of about 10° to avoid any parasitic oscillating and held by fiber chuck holders with U-shaped groove heat sinks. The pump beam was coupled into the active fiber by a coupling system consisted of a collimator (L1, *f* = 11 mm) and a CaF_2_ lens (L2, *f* = 15 mm). To achieve passive mode-locking, a piece of commercial SESAM (BATOP GmbH) was used as saturable feedback of the cavity. Follows are some typical parameters of the SESAM (provided by the manufacturer). The high reflection band is 2000 ~ 3400 nm accompanied with a relaxation time of around 10 ps. The absorbance and modulation depth are 33% and 18%, respectively, corresponding to a non-saturable loss of 15%. While the saturation fluence and damage threshold are 70 μJ/cm^2^ and 350 MW/cm^2^, respectively. The laser beam was collimated and then focused onto the SESAM through a couple of uniform CaF_2_ lenses (L3 and L4) with a focal length of 12 mm.

To make the fiber laser tunable and improve the stability of mode-locked pulse output, a blazed grating was placed in the other side of the cavity. The specifications of the grating are grooves of 625 mm^−1^, blaze wavelength of 2.8 μm and blaze angle of 61.2°. The laser beam was collimated by L2 and then reflected onto the grating by a dichroic mirror (M1, high reflection > 99.5% at 2.8 μm, high transmission > 95% at 975 nm). A piece of 2 mm thick Ge plate was placed with an incidence angle of 45° between the dichroic mirror M1 and the grating to couple out the laser beam. The reflectivity of Ge plate at 45° angle was measured to be around 55%, which was also the coupling ratio of the output 1. Taking the reflectivity of the grating into account, the other portion from the output 2 was around 22%. Consequently, the total output coupling ratio was ~77%. The total length of the laser cavity is about 3.49 m (i.e., the fiber length of 3.36 m and free space length of 0.15 m), leading to a repetition rate of 28.9 MHz (*f*_rep_ = *c*/2*L*_eff_, where *L*_eff_ = *n*_fiber_ *L*_fiber_ + *L*_freespace_). The group velocity dispersion of the ZBLAN fiber is *β*_2_ = −0.086ps^2^/m at 2.8 μm, therefore, the total dispersion of the cavity is around −0.29 ps^2^.

## Conclusion

In summary, we have demonstrated a tunable mode-locked ZBLAN fiber laser operating at about 2.8 μm for the first time to the best of our knowledge. A SESAM and a blazed grating in Littrow configuration were used to construct the linear cavity and served as the absorber and the wavelength tuning element, respectively. The maximum average output power was over 200 mW with a repetition rate of 28.9 MHz and a pulse duration of 6.4 ps at 2770.8 nm, yielding a maximum peak power of ~1.1 kW. The wavelength of the CML fiber laser was tunable over 100 nm from 2710 to 2820 nm through tuning the grating. The laser is potentially an ideal pulsed seed source for amplification to generate supercontinuum at mid-infrared region.
